# Efficacy of an Oral Solution Prepared from the Ultrasonic Extract of *Radix dichroae* roots against *Eimeria tenella* in Broiler Chickens

**DOI:** 10.1155/2020/3870902

**Published:** 2020-05-30

**Authors:** Ling Wang, Zhiting Guo, Zhenxing Gong, Jianping Cai, Feng Yang, Xiaojuan Wei, Biao Niu

**Affiliations:** ^1^Key Laboratory of New Animal Drug Project, Gansu Province/Key Laboratory of Veterinary Pharmaceutical Development, Ministry of Agriculture and Rural Affairs/ Lanzhou Institute of Husbandry and Pharmaceutical Sciences of Chinese Academy of Agriculture Sciences, Lanzhou 730050, China; ^2^State Key Laboratory of Veterinary Etiological Biology/Lanzhou Veterinary Research Institute of Chinese Academy of Agricultural Sciences, Lanzhou 730046, China

## Abstract

This study was conducted to determine the optimal dose of the oral solution of the ultrasonic extract of *Radix dichroae* (UERD) and to provide experimental support for a safe clinical dose for anticoccidial treatment of broiler chickens. *Radix dichroae* root extracts were prepared using the ultrasonic extraction method. The anticoccidial activity of the oral solution prepared from the ultrasonic extract of *Radix dichroae* roots was tested in broiler chickens following oral infection with a field isolate of *E. tenella*. Ninety Lingnan yellow broiler chickens (14 days old) were randomly divided into nine groups (*n* = 10), including six UERD oral solution treatments (0.25, 0.50, 1.50, 2.50, 3.50, and 5.00%), a toltrazuril group (0.10%), an *E. tenella*-infected control group, and a healthy control group. All groups were inoculated orally with 7 × 10^4^ sporulated *E. tenella* oocysts (Guangdong strain) except for the healthy control group. The chickens in the seven drug-treated groups were administered a UERD oral solution or toltrazuril in drinking water for 7 days. The anticoccidial efficacy of the UERD oral solution was evaluated by the bloody diarrhoea severity level, relative body weight gain (rBWG), lesion score, oocyst per gram (OPG), and anticoccidial index (ACI). Compared with the infected control group, there were no significant differences in the groups treated with UERD oral solution or toltrazuril with regard to the lesion changes in the caecal regions (*P* > 0.05); however, the blood contents, OPG, and oocyst score in three UERD oral solution treatment groups (0.50, 1.50, and 2.50%) were significantly reduced, and the bloody diarrhoea was also alleviated. The ACI in three UERD oral solution treatment groups (0.50%, ACI = 143.7; 1.50%, ACI = 151.0; and 2.50%, ACI = 144.3) was higher than that in the toltrazuril group (ACI = 127.0), and the rBWG in the 1.50% UERD oral solution treatment group (95.0%) was similar to that in the healthy control group (100%), which was also 12.5% higher than that in the toltrazuril group (82.5%). The findings of this study demonstrated that the UERD oral solution (0.50% ～ 2.50% dose range) showed better prevention, anticoccidial efficacy, and growth promotion effects than toltrazuril (0.10%), and the 1.50% dose level of UERD oral solution in water is the clinically recommended dose according to the present study conditions.

## 1. Introduction

Chicken coccidiosis is an intestinal disease caused by intracellular protozoan parasites belonging to the genus *Eimeria*, and the most severe harm to chickens is always caused by *Eimeria tenella* (*E. tenella*) [1–3], resulting in extensive destruction of the caecal epithelium, haemorrhagic faeces, reduced body weight gain, and feed conversion, as well as increased morbidity and mortality. Herbal products have been effectively used for the control and treatment of ailments in poultry and humans. *Radix dichroae* (RD) is the dried root of *Dichroa febrifuga* Lour., which belongs to the family of Saxifragaceae and is widely distributed in humid mountainous and other subtropical areas, such as the Sichuan, Guizhou, and Yunnan provinces in China. *Radix dichroae* is a famous herb and has been traditionally utilized as an antimalarial, expectorant, and antifebrile compound in China for centuries according to previous reports by Zhang and Huang [4] and Kirandeep et al. [5]. The analytical data revealed that *β*-dichroine and *α*-dichroine were the main anticoccidial effective components of *Radix dichroae*, with *β*-dichroine having more effective pharmacological activity than *α*-dichroine [6, 7]. Furthermore, halofuginone hydrobromide is a synthetic analogue of *β*-dichroine and has been used as an antiparasitic feed additive for the prevention of coccidiosis in poultry production, which has been approved by the European Union and the US Food and Drug Administration [7–9]. However, the use of halofuginone in the clinic has been restricted due to the complexity of the synthetic route and the high synthetic cost. Unfortunately, with the widespread use of chemoprophylaxis and anticoccidial feed additives, coccidiosis has been controlled but accompanied by the emergence of drug resistance [10, 11] and toxic effects on animal health [12]. Moreover, drug or antibiotic residue in poultry products is potentially troublesome to the consumer. Therefore, alternative strategies are being sought for more effective and safe control against chicken coccidiosis, which has become a top priority for the poultry industry [13, 14], and phytotherapies have been investigated as alternative methods for controlling coccidial infections, with which a number of herbal extracts have been indicated to be effective in controlling coccidiosis. *Radix dichroae* has anticoccidial properties in chickens when used as a single herb or as the main herb in an herbal formulation complex during treatment; however, the content of the active ingredients (i.e., the total alkaloids and *β*-dichroine) in the crude extract of *Radix dichroae* in the above coccidiostats (single *Radix dichroae* or *Radix dichroae* complex) has always been lower. Therefore, in this study, the total alkaloids as active ingredients were extracted from the dried roots of *Radix dichroae* using a modified phytomedicine extraction separation technique to increase the content of *β*-dichroine therein. Moreover, considering that the constituents in the oral solution could more easily access and interact with the parasites than the constituents remaining in the powder solid, the production technique was optimized to prepare an oral solution with the ultrasonic extract of *Radix dichroae* (UERD) to improve the treatment effects and ease of use in clinical settings. Therefore, the objective of this study was to determine the potential anticoccidial activity of UERD oral solution; a pathological model of chicken coccidiosis was reproduced after *E. tenella* (Guangdong strain) was inoculated artificially into chickens to further explore the efficacy of the oral solution prepared with UERD, as well as to determine the optimal dose in clinical practice. Employing a standard parasitological procedure as an alternate phytomedicine and prescription would contribute to the control of chicken coccidiosis.

## 2. Materials and Methods

### 2.1. Oral Liquid Preparation of the Ultrasonic Extract of *Radix dichroae*

*Radix dichroae* roots were purchased from Lanzhou Huanghe Chinese medicine market and identified as the dried root of *Radix dichroae* in the genus *Bergenia* of the family Saxifragaceae by Professor Yun Li from Gansu University of Chinese Medicine. Voucher specimens were deposited in the Herbarium of the College of Pharmacy (Medicinal Plant Collection) for *Radix dichroae* (voucher number: GUCM 621222130517114 LY). Two hundred grams of dried *Radix dichroae* roots was ground into powder using an electric grinder, and the extraction of the *Radix dichroae* roots was carried out using an ultrasonic extraction method. Two percent hydrochloric acid was used as the extraction solvent, and the solid-liquid ratio was 1 : 6 (g/mL, w/v). Ultrasonic wave power was used at 80 W, for 1 h extraction at 50°C, and 3 extraction stages were used. The supernatants collected from each stage were pooled together, the impurities were removed with a small amount of chloroform, and then the pH value was adjusted to 10 with strong ammonia. The solution was extracted 3 times with chloroform, and the recovered chloroform extracts containing the extract of *Radix dichroae* were concentrated, dried, and ground into a powder. The end product was approximately 25.80 g of dried extract powder, for an overall yield of 12.90% (w/w). The average contents of total alkaloid and *β*-dichroine therein were 4.79% and 0.18%, respectively. The optimal production conditions for the oral solution preparation of UERD were 120 mg UERD powder dissolved in 100 mL of a solution, by a prepared solution of 1.5% Tween-80 and 0.5% sodium benzoate in distilled water (1.20 mg/mL, w/v) to obtain the test solution with a pH value of 5–6 that was stored at 4°C until use. The positive control drug was toltrazuril (2.5%, w/v, Bayer (Sichuan) Animal Health Co. Ltd, China), administered in drinking water at the recommended dose of 0.10% (v/v).

### 2.2. Experimental Animals

Ninety one-day-old Lingnan yellow broiler chickens obtained from the Hualong Commercial Hatchery of Lanzhou were reared in a coccidia-free isolation facility and fed commercial food that contained no anticoccidial drugs or antibiotics for 14 days ad libitum during the study under the following conditions: temperature (23 ± 2°C), relative humidity (55 ± 15%), and ventilation (air exchange rate of 18 cycles/h). To confirm that the chickens were free of infection prior to experimental inoculation, faecal samples were analyzed by salt flotation and light microscopy to ensure the absence of oocysts. All animal care procedures were performed in accordance with the Veterinary Laboratory Biosafety Guidelines (the Chinese Ministry of Agriculture Guide, 2012) [15]. The study protocol was approved by the Animal Research Ethics Committee of Lanzhou Institute of Husbandry and Pharmaceutical Sciences, Chinese Academy of Agricultural Sciences (CAAS).

### 2.3. Preparation of *E. tenella* Oocysts

The oocysts of *E. tenella* (Guangdong strain) used in this study were isolated from chickens that died from *E. tenella* infection in 1996 in Huadu, Guangdong province, China, as confirmed by microscopic examination and sequence analysis of the rRNA gene internal transcribed spacer regions, and maintained in the State Key Laboratory of Veterinary Etiological Biology, Lanzhou Veterinary Research Institute, CAAS. The oocysts were propagated in the broiler chickens without *E. tenella* infection by oral infection, and the faeces were collected on postinfection (PI) days 6, 7, 8, and 9. The unsporulated oocysts were sporulated by placement in a 2.5% potassium dichromate solution at a suitable humidity and temperature (28°C). Sporulated oocysts were cleaned with water and counted by the McMaster technique mentioned by Foreyt [16].

### 2.4. Study Design

A total of ninety Lingnan yellow broiler chickens (14 days old) free from coccidian infection were weighed individually and randomly divided into nine groups (*n* = 10). At 14 days of age, the chickens in groups IC (infected/positive control group; nontreated and *E. tenella* infected), DC (positive drug control group), A, B, C, D, E, and F were orally infected with 7.0 × 10^4^ sporulated *E. tenella* oocysts. The clinical signs were observed and recorded every day during the experiment. Signs of infection were defined as the presence of oocysts in the faeces with bloody diarrhoea according to the method of Holdsworth et al. [17]. At 15 days of age, all of the chickens except for those in group HC (healthy/negative control group; nontreated and noninfected) and group IC began drug treatment. The chickens in group DC (drug control group) were administered toltrazuril in drinking water at a dose of 0.10% (v/v) for 7 days. The chickens in groups A, B, C, D, E, and F were treated with six UERD oral solutions, which were administered in water at doses of 0.25, 0.50, 1.50, 2.50, 3.50, and 5.00% (v/v) for 7 days. Each group was allocated to a large cage with a single tray per group to catch the faecal material. At 22 days of age, after weighing the surviving chickens individually, all of the chickens were sacrificed humanely for the grading of the caecal lesions, and the survival rate was calculated for each group.

### 2.5. Data Collection

#### 2.5.1. BW Gain and Faecal Score

Each chicken was weighed on day 14 before *E. tenella* infection and at the end of the experiment (8 days after infection); additionally, all surviving chickens were weighed individually before culling. The individual and mean body weight gains were calculated for the period of days 14–22. The relative body weight gain (rBWG) was calculated using the following formulae: BWG = final body weight － initial body weight; BWG rate (%) = (final body weight － initial body weight) ÷ initial body weight × 100%; rBWG (%) = (BWG rate of the infected control or drug-treated group ÷ BWG rate of healthy control group) × 100%. Clinical observations of bloody diarrhoea and mortality for all the chickens were recorded daily throughout the experimental period. The survival rate (%) = (number of surviving chickens in each group ÷ number of initial chickens in each group) × 100%. Faecal droppings were examined visually for bloody diarrhoea during the days 4–7 after infection according to the method of Morehouse and Baron [18] and assigned to one of five ranks (0, 1, 2, 3, 4) based on the evaluation standard of Suo and Li [19]. The ranks are as follows: 0 (normal), no bloody samples; 1, 25% piles of bloody diarrhoea; 2, 50% piles of bloody diarrhoea; 3, 75% piles of bloody diarrhoea; 4, 4 or more piles of bloody diarrhoea.

#### 2.5.2. Oocyst Counts and Caecal Lesion Score

On days 5, 6, and 7 after infection, the total daily faeces of each group were collected, and the daily oocyst production was determined using a McMaster chamber [16]. The surviving chickens were euthanized on postinfection day 8, and the lesion scores of the caecum were examined and evaluated immediately using the lesion scoring technique of Johnson and Reid [20]. The grade scoring system (from 0 to 4) was adopted. The lesion score = the average lesion score in each group × 10.

Then, the caecal contents were ground, and the oocyst counts in the pooled caecum contents of each chicken were determined using the McMaster's method. The result was expressed as oocysts per gram (OPG) based on the method of JIAO [21]. The oocyst values were calculated based on the following OPG values: an OPG ≤0.1 gave an oocyst value of 0, an OPG between 0.1 and 1.0 gave an oocyst value of 1, an OPG between 2.0 and 5.0 gave an oocyst value of 10, an OPG between 6.0 and 10.0 gave an oocyst value of 20, and an OPG ≥11.0 gave an oocyst value of 40.

The anticoccidial index (ACI), established by Merck Sharp & Dohme [22], was calculated as ACI = (rBWG + survival rate) × 100 − (lesion score + oocyst value).

### 2.6. Statistical Analysis

The data were analyzed using SPSS 17.0 software (SPSS Inc., Chicago, IL, USA). The bloody diarrhoea was statistically compared by the Kruskal-Wallis H test, and the results were expressed as median frequencies (interquartile range, 95% confidence interval). The parameters of mean body weight for each time point, body weight gain, and mean lesion score were conducted using one-way analysis of variance, followed by a LSD (least significant difference) test, Dunnett's post hoc test, or Duncan's multiple range test, which were used to test the significance between different groups at the level of *P* < 0.05. The results are expressed as the means ± SD.

## 3. Results

### 3.1. Clinical Signs and Bloody Diarrhoea

Once infected with sporulated *E. tenella* oocysts, chickens took 3 days to demonstrate clinical symptoms of infection to different degrees, including depression, loss of appetite, drowsiness, feather disorders, fear of cold, and huddling. Bloody diarrhoea was observed in all infected groups from postinfection day 4 to 7. The most severe bloody diarrhoea was found in group IC (infected/nontreated group), which had the highest bloody score than that of any of the other groups (bloody score (median) = 4, *P* < 0.05), followed by group A (bloody score (median) = 4, *P* < 0.05), group F (bloody score (median) = 2), and the toltrazuril group (bloody score (median) = 2). The degree of bloody diarrhoea and faecal score in four of the groups treated with the UERD oral solution (0.50, 1.50, 2.50, and 3.50%) were lighter and milder among the 7 drug-treated groups, and the bloody score in the 5.00% UERD oral solution group was similar to that in the toltrazuril group. The broiler chickens administered UERD oral solution and toltrazuril showed lower bloody scores than those in the infected/nontreated group (<3 points, except group A), and the physical condition and feed intake improved obviously. Three chickens in the positive infected control group (group IC) died from coccidial infection; and in the 0.10% toltrazuril group and four UERD oral solution treatment groups (0.25, 1.50, 3.50, and 5.00%), one chicken died from *E. tenella* infection in each group (Table 1).

### 3.2. Pathologic Anatomy, OPG, and Oocyst Counts

On the 8^th^ day after inoculation, all surviving chickens were humanely sacrificed for the grading of caecal lesions, and the results of lesion scores are shown in Table 2. Chickens in the positive infected control group (group IC) displayed significant swelling in the caecal regions and contained a large amount of blood sample contents, and in the duodenal regions bleeding spots and points were found. Intestinal damage was also found in all chickens in the experimental groups; however, the degree of intestinal lesions in six of the UERD oral solution treatment groups and the toltrazuril treatment group was lower than that of the infected control group. No obvious lesions in other organs were found, and only a few infected chickens showed severe macroscopically visible lesions. Among the 7 drug-treated groups, the lesion scores of group DC administered 0.10% toltrazuril were the highest; however, there was no significant difference in lesion scores between the 6 UERD-treated groups and the toltrazuril control group.

As shown in Table 2, except for the healthy control group (group HC), all the chickens in the UERD oral solution and toltrazuril treatment groups showed a reduction in the oocyst counts. The OPG and oocyst output in the infected control group (group IC, OPG = 6.79 × 10^6^) were the highest among groups infected with sporulated *E. tenella* oocysts. The group medicated with 0.10% toltrazuril (group DC) had the lowest output of oocysts with an OPG of 3.11 × 10^6^. The OPG and oocyst values in groups B, C, and D (0.50, 1.50, and 2.50% UERD oral solution) and group DC (0.10% toltrazuril) were significantly lower than those in group IC (infected/nontreated group). However, chickens administered UERD oral solution at doses of 3.50% and 5.00% showed similar OPG and oocyst values to those of the infected control group (group IC).

### 3.3. Growth Promotion

The rBWG of group C administered 1.50% UERD oral solution (95.0%) was similar to that of group HC (100%), which was 12.5% higher than that of group DC (0.10% toltrazuril; 82.5%). The chickens in the groups treated with 0.50, 1.50, and 2.50% UERD oral solution and 0.10% toltrazuril had no significant difference in the mean final weights compared with group HC, while there was a significant difference compared with group IC (*P* < 0.05). Moreover, chickens in group F treated with 5.0% UERD oral solution had a similar and lower rBWG compared with group IC.

### 3.4. Anticoccidial Index (ACI)

The ACI values in the six UERD oral solution treatments and toltrazuril treatment were all higher than the infected control group (group IC). The highest ACI value was observed in group C (1.50% UERD oral solution, ACI = 151.0), followed by group D (2.50% UERD oral solution, ACI = 144.3) and group B (0.50% UERD oral solution, ACI = 143.7). Moreover, the ACI values in these three UERD-treated groups were superior to toltrazuril treatment (ACI = 127.0). However, with the increasing dose of UERD oral solution, the ACI values in group E (3.50% UERD oral solution, ACI = 118.0) and group F (5.00% UERD oral solution, ACI = 111.1) decreased significantly, and the lowest ACI value from the six UERD-treated groups was observed in group A (ACI = 99.1) (Table 2).

## 4. Discussion

Coccidiosis is one of the biggest challenges faced by the global poultry industry and results in annual losses, including decreased productivity and the usage of anticoccidial drugs and vaccines, estimated at approximately 3 billion US dollars worldwide [13, 23, 24]. At present, chemoprophylaxis and anticoccidial feed additives are used to control the spread of coccidiosis, in addition to the chemical synthesis of drugs with excellent efficiency to prevent and treat chicken coccidiosis, including diclazuril, toltrazuril, and halofuginone. Many studies have been conducted on the anticoccidial effects and mechanism of the above-mentioned drugs [25]. However, these methods have become complicated by the emergence of drug resistance and the toxic effects of such additives on animal health [10–12]. Furthermore, drug residue in poultry products is a potential constraint and may be harmful to the consumer. To date, the anticoccidial activity of 32 plants and 40 phytocompounds has been scientifically evaluated [26, 27]. As a result of different sources from traditional Chinese medicines (TCMs) and chemical synthetic drugs, the function, mechanism, and therapy theory between these two classes of drugs differ greatly in the processes of prevention and control of chicken coccidiosis; moreover, TCM is usually characterized as presenting multitarget, multichannel, and synergistic effects against diseases. According to the theory of evidence-based medicine, the subordinate syndrome of chicken coccidiosis developed from an exogenous affection to damp heat, where the spleen showed dysfunction immediately after the damp heat accumulation in the large intestine. This resulted in acute massive haemorrhage forced by heat toxicity and expressed bloody diarrhoea, so the treatment principle for chicken coccidiosis should give priority to eliminate the heat and dampness, which will cool the blood and stop the diarrhoea. *Radix dichroae* is a famous herb used as an antimalarial, expectorant, and antifebrile medicine in Chinese medicine for centuries, and its antimalarial pharmacological activities were researched by Jang et al. in 1948 [28]. Moreover, it also has anticoccidial effects in chickens infected by coccidiosis when it is used alone or as the main herb in a complex formulation. The findings from previous studies indicated that the crude extract of *Radix dichroae* and the *β*-dichroine found therein could promote the proliferation of spleen T lymphocytes and B lymphocytes and had better immune-enhancing activity for macrophages in mice [9]. Furthermore, the results from the toxicology test by Wang et al. (2018) demonstrated that the ultrasonic extract of *Radix dichroae* roots possessed lower toxicity and showed dose-dependent toxicity in the liver, kidneys, spleen, and lungs at high doses after a long course of administration [29]. Hence, the aim of this study was to investigate the anticoccidial activity of an oral solution prepared from the ultrasonic extract of *Radix dichroae* (UERD) in chickens to determine whether UERD oral solution can be used as an effective and safe Chinese medicine under conventional dose conditions and also to determine if it is suitable for long-term clinical use against coccidiosis.

Considering that poultry has a different gastrointestinal structure compared with ruminants, it is impossible for chickens to completely digest and absorb the crude extract of *Radix dichroae*. Additionally, it easily causes gastrointestinal dysfunction, which in turn greatly reduces its anticoccidial effect, causing substantial waste of the original medicinal materials. Therefore, the development of an oral suspension for the ultrasonic extract of *Radix dichroae* can ensure the utilization of the highest drug content and meet the fluidity requirements for oral administration. Second, oral solution preparation has the advantages of stable quality, fast absorption, a good curative effect, and a safe and hygienic clinical application, which is convenient for absorption and utilization in chickens and thus can better exert anticoccidial activity. *E. tenella* is the most pathogenic chicken coccidian and mainly parasitic in the caecal region, where active infection is characterized by diarrhoea and massive caecal haemorrhage. Infection with *E. tenella* typically leads to swelling and damage to the caecal wall, which alters the normal function of the chicken intestine [30]. In this study, bloody diarrhoea was prolonged and severe in the infected control group, especially on postinfection (PI) days 4, 5, 6, and 7 (Table 1), which are the most important days in the lifecycle of *E. tenella* and when three chickens died of coccidiosis infection. Signs of infection were alleviated by drinking water supplementation with UERD oral solution and toltrazuril. Compared with the infected control group, the degree of severity of the caecal lesion was significantly improved in groups medicated with UERD oral solution and toltrazuril, and the oocyst value and OPG were both reduced in the drug-treated groups (except for the 5.00% UERD oral solution treatment group). The findings in this study indicated that UERD oral solution at doses of 0.50, 1.50, and 2.50% in broiler chickens could significantly increase the rBWG compared with that in the infected control group after challenge with sporulated *E. tenella*. In addition, the amount of bloody faeces, the OPG, and the oocyst score also improved in chickens after administering UERD oral solution at the recommended dose mentioned above. It can be confirmed that the ultrasonic extract from a single herbal *Radix dichroae* root extract had a curative effect against *E. tenella* infection in broiler chickens. However, chickens medicated with the 0.25% and 5.00% UERD oral solutions tended to have a lower rBWG compared with the other drug-treated groups, while having a similar rBWG to the infected control group; this reduced rBWG might be inferred as the effect caused by the lower doses of UERD oral solution, which contain inadequate amounts of the active ingredients (such as *β*-dichroine). However, for the higher dose of UERD oral solution, the toxic reaction from excessive active ingredients might hamper the growth of the chickens and cause high OPG values (mentioned above).

The lesion score, OPG, and ACI values are commonly applied parameters used to evaluate the anticoccidial efficacy of animal drugs. In this study, the highest lesion score and lowest ACI value were observed in the infected control groups, followed by the 0.25%, 3.50%, and 5.00% UERD oral solution treatments, which showed lower lesion scores and higher ACI values. Moreover, similar lesion scores and oocyst values but higher ACI values were observed in 3 UERD oral solution treatment groups (0.50, 1.50, and 2.50%) and in the 0.10% toltrazuril treatment group, in which 0.10% toltrazuril had lower OPG and ACI values. These results indicated that the UERD oral solution and toltrazuril produced better protective effects on the intestinal mucosa, and the concentration of UERD oral solution within the range of 0.50% ～ 2.50% demonstrated better anticoccidial efficacy than 0.10% toltrazuril in the present study. In addition, although the anticoccidial effects of the two highest UERD oral solution treatments (3.50% and 5.00%) were better than those of the *E. tenella-*infected control group, the UERD oral solution administered by drinking water at high doses might produce drug toxicity, which would be harmful to chickens in the clinic. In this study, the ACI values from all the drug-treated groups were all less than 160, which may be related to the virulence of the *E. tenella* strain selected in the test (Guangdong strain). The field isolate of *E. tenella* (Guangdong strain) obtained from the State Key Laboratory of Veterinary Etiological Biology has been used in research on chicken coccidiosis for a long time by Li et al. [31]. The virulence of this field isolate is stronger, and the mortality caused by it ranged from 10% to 20% after the chickens were inoculated with 7 × 10^4^ oocysts of sporulated *E. tenella* within one month. In general, mortality ranging from 5% to 10% in the infection control group is considered optimal in the experimental design for anticoccidial drugs. However, in this study, after challenge with 7 × 10^4^ oocysts of sporulated *E. tenella* (Guangdong strain), the mortality rate in the infection control group reached 30%, and we can infer from this result that the chickens in eight drug-treated groups tended to be inoculated with a relatively high dose of sporulated *E. tenella* oocysts. Although lower ACI values were expressed in the toltrazuril and six UERD oral solution treatments in the present study, the relationship between the drug dose and the anticoccidial effect could still be obtained from the results; that is, the UERD oral solution administered through drinking water according to the recommended doses of 0.50% (ACI = 143.7), 1.50% (ACI = 151.0), and 2.50% (ACI = 144.3) had better anticoccidial effects than 0.10% toltrazuril (ACI = 127.0). Considering the drug cost in the clinic and the growth performance of broiler chickens, 1.50% UERD oral solution in drinking water should be the clinically recommended dose for broiler chickens.

## 5. Conclusion

In this study, a higher protection rate was found in three UERD oral solution treatments (0.50, 1.50, and 2.50%) compared with the 0.10% toltrazuril treatment. The addition of the UERD oral solution could increase the body weight and survival rate of broiler chickens infected with *E. tenella* (Guangdong strain) while reducing bloody diarrhoea, oocyst output, and lesion scores in the caecal region. It was demonstrated that UERD oral solution within a 0.50% ～ 2.50% dose range had better anticoccidial and growth promoting effects than toltrazuril in chickens infected with sporulated *E. tenella* oocysts. The oral solution prepared with the ultrasonic extract of *Radix dichroae* roots was suitable for administration in drinking water, and its good efficacy after 7 days of treatment revealed that the UERD oral solution was appropriate for the prevention, therapy, and intermittent treatment of *E. tenella-*infected chickens.

## Figures and Tables

**Table 1:** Bloody diarrhoea and mortality in broiler chickens during days 4–7 after infection (*n* = 10).

Groups Drug level (v/v,%)	Deaths	Mortality (%)	Postinfection days				Total bloody faecal	Bloody scores (median)
			4^th^	5^th^	6^th^	7^th^		
Group HC nontreated and noninfected Healthy control (HC)	0	0	0	0	0	0	0	0^a^
Group IC nontreated and infected Infected control (IC)	3	30	3.6	4.0	4.0	4.0	15.6	4^c^
Group DC 0.10% toltrazuril Positive drug control (DC)	1	10	2.8	2.8	2.0	1.2	8.8	2^ba^
Group A 0.25% UERD oral solution	1	10	3.0	3.0	2.8	2.8	11.6	4^c^
Group B 0.50% UERD oral solution	0	0	2.4	2.0	2.0	1.0	7.4	0^a^
Group C 1.50% UERD oral solution	1	10	2.4	2.4	1.6	1.6	8.0	0^a^
Group D 2.50% UERD oral solution	0	0	2.4	2.0	1.6	1.6	7.6	0^a^
Group E 3.50% UERD oral solution	1	10	2.4	2.4	2.0	2.0	8.8	0^a^
Group F 5.00% UERD oral solution	1	10	2.8	2.8	2.4	2.4	10.4	2^ba^

UERD: ultrasonic extract of *Radix dichroae*. Columns with different superscripts present significant differences (*P* < 0.05).

**Table 2:** Effects of UERD oral solution on oocyst output, lesion score, and anticoccidial activity in broiler chickens against *E. tenella* (Guangdong strain) (*n* = 10).

Groups	Initial body weight (g)	Final body weight (g)	BWG (g)	BWG rate (%)	rBWG (%)	Survival rate (%)	Mean lesion score	Lesion score	OPG (×10^6^)	Oocyst value	Anticoccidial index (ACI)
Drug level (v/v,%)											
Group HC nontreated and noninfected Healthy control (HC)	247.9 ± 16.8^a^	366.7 ± 24.0^b^	118.8 ± 12.4^b^	48.0	100	100	0	0	0	0	200
Group IC nontreated and infected Infected control (IC)	239.6 ± 22.6^a^	319.4 ± 15.9^a^	78.6 ± 8.0^a^	32.9	68.5	70	3.85 ± 0.21^a^	38.5	6.79	20	80.0
Group DC 0.10% toltrazuril Positive drug control (DC)	244.9 ± 23.1^a^	347.3 ± 18.9^b^	98.0 ± 8.8^b^	39.6	82.5	90	3.55 ± 0.46^a^	35.5	3.11	10	127.0
Group A 0.25% UERD oral solution	256.4 ± 24.3^a^	316.3 ± 29.5^a^	64.3 ± 11.1^a^	25.5	53.1	90	3.40 ± 045^a^	34.0	5.22	10	99.1
Group B 0.50% UERD oral solution	251.3 ± 19.7^a^	355.9 ± 34.2^b^	104.6 ± 19.9^b^	41.6	86.7	100	3.30 ± 0.38^a^	33.0	4.30	10	143.7
Group C 1.50% UERD oral solution	255.7 ± 24.6^a^	371.2 ± 26.5^b^	115.5 ± 9.22^b^	45.6	95.0	100	3.40 ± 0.36^a^	34.0	4.44	10	151.0
Group D 2.50% UERD oral solution	247.8 ± 20.2^a^	352.3 ± 40.2^b^	104.5 ± 21.6^b^	41.9	87.3	100	3.30 ± 0.49^a^	33.0	4.80	10	144.3
Group E 3.50% UERD oral solution	248.7 ± 18.8^a^	339.4 ± 31.0^a^	94.3 ± 19.9^b^	38.4	80.0	90	3.20 ± 0.45^a^	32.0	6.11	20	118.0
Group F 5.00% UERD oral solution	252.9 ± 17.5^a^	336.2 ± 25.2^a^	85.7 ± 14.8^a^	33.9	70.6	90	2.95 ± 1.20^a^	29.5	8.11	20	111.1

^a-b^Means ± SD; means with different superscripts in the same column differ significantly (*P* < 0.05). BWG: body weight gain; rBWG: relative body weight gain; OPG: oocysts per gram; UERD: ultrasonic extract of *Radix dichroae*

## Data Availability

The data used to support the findings of this study are available from the corresponding author upon request.
